# Antioxidant synergistic effects of *Osmanthus fragrans* flowers with green tea and their major contributed antioxidant compounds

**DOI:** 10.1038/srep46501

**Published:** 2017-04-19

**Authors:** Shuqin Mao, Kaidi Wang, Yukun Lei, Shuting Yao, Baiyi Lu, Weisu Huang

**Affiliations:** 1 Zhejiang University, College of Biosystems Engineering and Food Science, Fuli Institute of Food Science, Zhejiang Key Laboratory for Agro-Food Processing, Zhejiang R & D Center for Food Technology and Equipment, Key Laboratory for Agro-Food Risk Assessment of Minstry of Agriculture, Hangzhou 310058, China; 2 Zhejiang Economic & Trade Polytechnic, Department of Applied Technology, Hangzhou 310018, China

## Abstract

The antioxidant synergistic effects of *Osmanthus fragrans* flowers with green tea were evaluated, and their major antioxidant compounds contributed to the total amount of synergy were determined. The antioxidant compounds in *O. fragrans* flowers with green tea were identified by LC-MS and quantified by UPLC-PDA. The synergistic antioxidant interactions between *O. fragrans* flowers with green tea and their antioxidant compounds were tested using the Prieto’s model after the simulated digestion. The main antioxidant compounds in *O. fragrans* flowers were acteoside and salideroside, whereas the main antioxidant compounds in green tea were caffeine, gallic acid, and L-epicatechin. The significant synergistic effect between *O. fragran*s flowers and green tea was observed and among nearly all of the combinations of their antioxidant compounds. Among the combinations, acteoside and gallic acid contributed most to the antioxidant synergy between *O. fragrans* flowers and green tea. However, the simulated digestion decreased this antioxidant synergy because it reduced the contents and the antioxidant capacities of their compounds, as well as the antioxidant synergy among the compounds.

The interaction among antioxidants is increasingly acknowledged because of its antioxidant effects on food stability and possible health benefits^1^. Antioxidants from natural sources, such as green tea extracts, contain several compounds with antioxidative properties. The antioxidants in these extracts interact with the antioxidants of lipid-containing food and with the endogenous antioxidants present in the food^2^. When the antioxidants are combined, antioxidant synergy occurs; this process generates an overall effect greater than the consolidated but separate antioxidant effects^3^.

*Osmanthus fragrans* is a common Chinese ornamental plant and used as a traditional folk medicine for thousands of years^4^. *O. fragrans* flowers contain several bioactive components, such as flavonoids and phenolic acids^5^, which exhibit neuroprotection, free radical scavenging, protection against aging and antioxidant effects^6^. In China, *O. fragrans* flowers are persistently used to improve the flavour of tea, food and beverages^7^. Recently, the antioxidant synergy among phenolic compounds, such as caffeic acid, gallic acid and chlorogenic acid, and flavonoids, such as rutin and quercetin, have been investigated *in vitro*^8^. Tea polyphenols are the most potent antioxidants among all known plant phenols^9^. Furthermore, they are reported to have antioxidant synergy with many substances, such as iron^10^, quercetin 3-β-glucoside^11^ and some herbs^12^. Colon and Nerín^13^ found that the flavonols in tea extracts provided additive interactions and the tea catechins contributed to the antioxidant synergy in green tea. Therefore, antioxidant synergy among the major antioxidant compounds in *O. fragrans* flowers and tea could be present.

However, the type of interactive effects is often determined through extremely simple relationships and through basic procedures instead of classical approaches^14^. Recently, a procedure has been described to identify and quantify the interactive effects between two antioxidants and applied to investigate interactive mechanisms in complex mixtures of antioxidants^15^. Furthermore, digestion plays an essential role in the effects of natural antioxidants in the human body. The total phenylethanoid glycoside contents and antioxidant activities in *O. fragrans* flowers were reported to decrease significantly after digestion^16^. Moreover, other studies showed that the total antioxidant capacity in tea extracts decreased after digestion *in vitro*^17^.

Thus, in this study, we aimed to measure the antioxidant synergy between *O. fragrans* flowers and green tea as well as among the antioxidant compounds in *O. fragrans* flowers with green tea using a simulated digestion model. Consequently, the major compounds which have the most contribution to antioxidant synergy were identified. We also aimed to analyse the influence of simulated digestion and examine the factors involved in this influence.

## Results and Discussion

### Antioxidant synergy of *O. fragrans* flowers and tea

**Figure 1:**
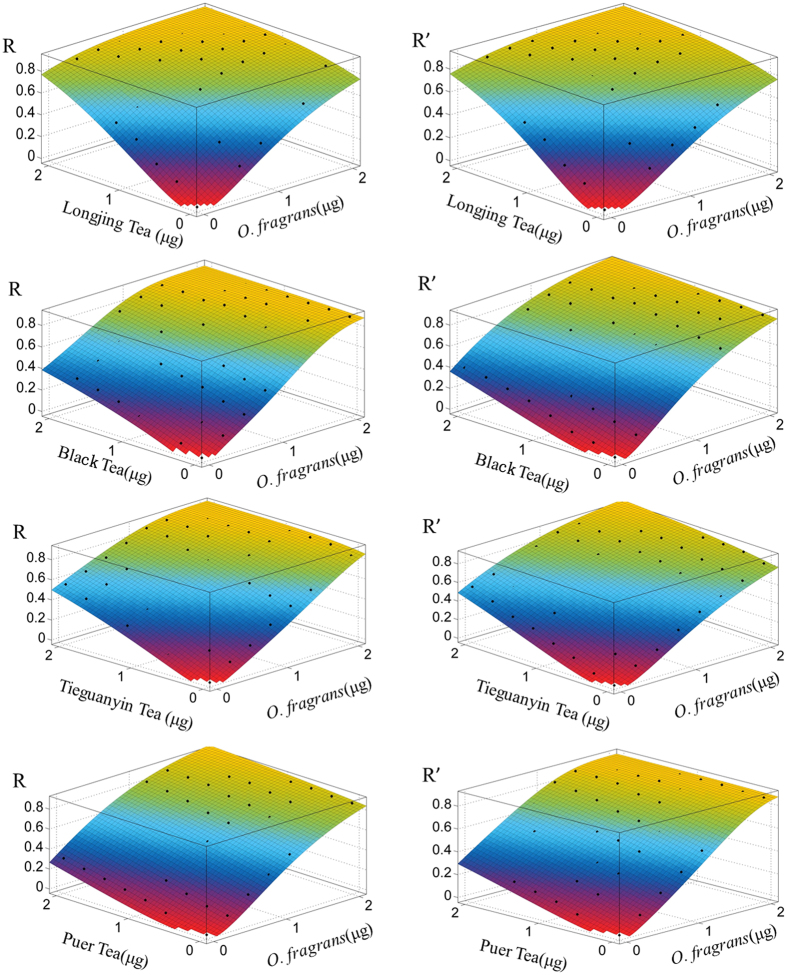
Antioxidant synergy of *O. fragrans* flowers and tea. Surface responses of joint effect between *O. fragrans* flowers and each of 4 teas in DPPH assay under different hypotheses. R: radical scavenging activity in DPPH antioxidant assay fitted into interaction model Eq. (4). R’: radical scavenging activity in DPPH antioxidant assay fitted into null interaction model Eq. (3). Response surfaces consist of experimental results (points) and fittings to the models (surfaces). The RUV values between *O. fragrans* flowers with Longjing tea, Pu’er tea, Black tea and Tieguanyin tea were 0.23%, 0.16%, 0.04% and 0.12%, respectively.

The antioxidant interactions between *O. fragrans* flowers and each of the four kinds of tea including Longjing Tea, Pu’er Tea, Black Tea and Tieguanyin Tea were investigated using the Prieto’s model. The results showed that *O. fragrans* flowers had synergistic antioxidant effects with all the teas (Fig. 1). Among all the combinations, the combination of *O. fragrans* flowers with Longjing Tea (0.23%) showed the most potent antioxidant synergy, whereas the combinations of *O. fragrans* with Black Tea (0.16%), with Tieguanyin Tea (0.04%) and with Pu’er Tea (0.12%) had less potent antioxidant synergies. The antioxidant compounds and their quantities in different types of tea considerably varied because of the distinct manufacturing process of each tea type^18^ (Table S1). Thus, the major antioxidant compounds in *O. fragrans* flowers and green tea were selected for further investigation.

### Main antioxidant compounds in *O. fragrans* flowers and green tea

**Figure 2:**
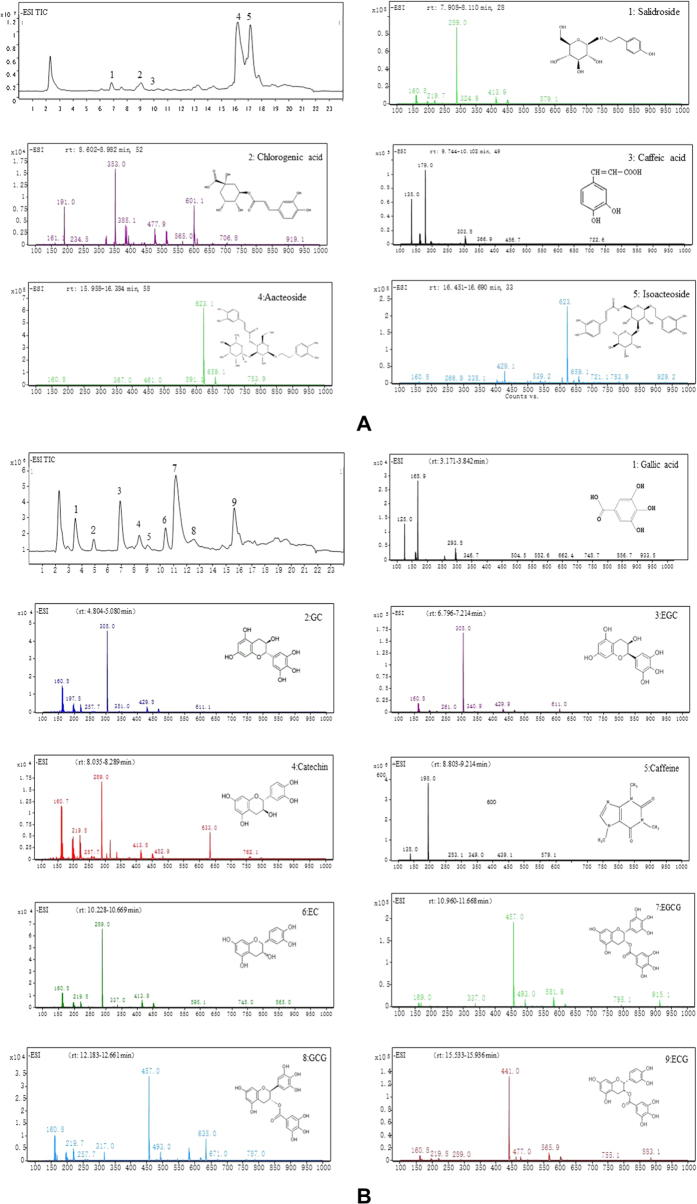
Total ion chromatogram of *O. fragrans* flowers (**A**) and green tea (**B**) and mass spectrum of their antioxidant compounds. (**A**) The first chart is the total ion chromatogram of water extract of *O. fragrans* flowers under the experimental conditions, and others are the mass spectrums of its antioxidant compounds. 1: salidroside 2: chlorogenic acid 3: caffeic acid 4: acteoside 5: isoacteoside. (**B**) The first chart is the total ion chromatogram of water extract of Longjing Tea under the experimental conditions, and others are the mass spectrums of its antioxidant compounds. 1: gallic acid 2: GC 3: EGC 4: catechin 5: caffeine 6: EC 7: EGCG 8: GCG 9: ECG.

According to the mass spectra of the antioxidant compounds presented in Fig. 2A, acteoside, salidroside, isoacteoide, chlorogenic acid and caffeic acid were identified and quantified in *O. fragrans* flowers, with contents of 10.77, 1.51, 0.39, 0.27 and 0.08 mg/g dry weight (DW), respectively. The same compounds were determined in various *O. fragrans* flowers using UPLC/PDA/MS after the simulated digestion^16^. Acteoside had the highest content among the antioxidant compounds in *O. fragrans* flowers, and this finding was consistent with that of Lu *et al*.^4^.

In green tea, the following antioxidants were identified and quantified: caffeine (10.86 mg/g), gallic acid (2.06 mg/g), EC (1.82 mg/g), GC (1.14 mg/g), EGC (1.12 mg/g), GCG (0.74 mg/g), catechin (0.54 mg/g) and ECG (0.19 mg/g). In previous studies, 10 polyphenol compounds (caffeine, gallic acid, GC, EGC, catechin, EGCG, EC, GCG, EGCG-3Me and ECG) in green tea leaves were identified through HPLC^19^. However, the average contents were all slightly higher than those in our study because their materials did not undergo simulated digestion.

However, EGCG, one of the most common compounds in green tea, was not detected. The reason was that EGCG underwent complex reactions during digestion and formed EC and an EGCG derivative with high molecular weight^20^. Thus, the EGCG content was below the quantification limit, as indicated in our results.

### Individual antioxidant capacity of *O. fragrans* flowers, green tea and their antioxidant compounds

The dose responses of *O. fragrans* flowers, green tea and their antioxidant compounds were fitted to model and the fitting parameters of the response procedure were shown in Table S2. The value of parameter m was equivalent to the EC_50_ in Table 1. Among all the antioxidant compounds, gallic acid showed the highest antioxidant capacity with an EC_50_ value of 3.52 μg/mL, whereas caffeic acid had the lowest with an EC_50_ value of 11.4 μg/mL.

Most compounds exhibited lower antioxidant capacity compared with the compounds in previous studies^21^. Meanwhile, acteoside and isoacteoside had higher DPPH radical scavenging activities compared with those in the results of Kim *et al*.^22^. The differences in the results were due to the simulated digestion, which resulted in the transformations and the losses of different antioxidant compounds to varying degrees.

In addition, salidroside and caffeine had poor DPPH free radical scavenging capacity, which could not fit the dose–response model well. Their antioxidant capacities were not determined by DPPH assay^23^. Therefore, they were excluded during the investigation of antioxidant interactions.

### Antioxidant interactions among antioxidant compounds of *O. fragrans* and green tea

**Figure 3:**
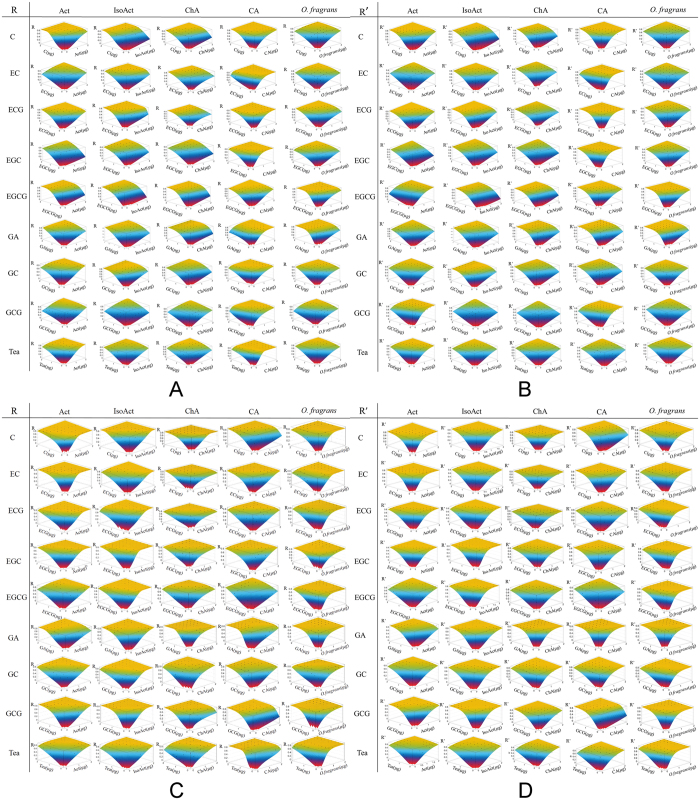
Antioxidant interactions among antioxidant compounds of *O. fragrans* flowers and green tea before (**A**,**B**) and after (**C**,**D**) the simulated digestion. Surface responses of joint effect for each pair of the 45 antioxidant combinations in DPPH assay under different hypotheses. R: radical scavenging activity in DPPH antioxidant assay fitted into interaction model Eq. (4). R’: radical scavenging activity in DPPH antioxidant assay fitted into null interaction model Eq. (3). Response surfaces consist of experimental results (points) and fittings to the models (surfaces). Numerical results of RUV were shown in Table 2. Act: actoside; IsoAct: isoacteoside; ChA: chlorogenic acid; CA: caffeic acid; C: catechin; EGCG: (−)-Epigallocatechin gallate; EC: L-Epicatechin; ECG: (−)-Epicatechin gallate; EGC: (−)-Epigallocatechin; GA: gallic acid; GC: (−)-gallocatechin; GCG: (−)-Gallocatechin gallate.

The surface responses in each pair of the 45 combinations were fitted to models, as shown in Fig. 3. Their RUV values were then quantified (Table 2). The global confidence interval of an RUV is [−0.17%, 0.33%]; thus, any RUV value outside this confidence interval is considered as statistically consistent. On the basis of this confidence limit, 16 combinations had significant synergistic interactions.

Among the 32 combinations of the 4 antioxidant compounds in *O. fragrans* flowers with the 8 antioxidant compounds in green tea, most showed synergistic antioxidant interactions. Particularly, 13 combinations had significant synergistic interactions, whereas 3 combinations had significant antagonistic interactions. The combination of caffeic acid and ECG showed the most effective antioxidant synergy with RUV of 0.85%, followed by the combination of chlorogenic acid and EC (0.77%). Among the 4 antioxidant compounds of *O. fragrans* flowers, chlorogenic acid showed the best synergistic effect with the antioxidant compounds in green tea as it had significant synergies with 6 green tea compounds, such as EC, GC, ECG, GCG, EGCG and EGC. By contrast, caffeic acid had the most antagonistic effects with the antioxidant compounds in green tea. It showed significant antagonistic effects with EGCG, GA and GC. Among the 8 antioxidant compounds in green tea, ECG had the best synergistic interactions with the antioxidant compounds in *O. fragrans* flowers. It exhibited significant synergy with caffeic acid, chlorogenic acid and isoacteoside. Meanwhile, the synergies among polyphenols and flavonoids were observed in previous studies. Palafox-Carlos *et al*.^24^ and Hajimehdipoor *et al*.^8^ revealed the antioxidant synergistic effects among chlorogenic acid, caffeic acid and gallic acid. In addition, de Kok *et al*.^25^ reported the general antioxidant synergies among EGCG, EC, EGC and ECG.

Among the 12 combinations between *O. fragrans* flowers with 8 antioxidant compounds in green tea and green tea with 4 antioxidant compounds in *O. fragrans* flowers, 3 combinations showed significant synergistic effects. Among the 4 antioxidant compounds of the *O. fragrans* flowers, chlorogenic acid and isoacteoside had significant synergistic effects with green tea. Among all the compounds in green tea, GCG showed the best synergy with *O. fragrans* flowers, with an RUV value of 0.43%. Meanwhile, Colon and Nerín^13^ observed that flavonols, such as quercetin and kaempferol, had synergistic effects with individual catechins, as well as the tea extract. Thus, the synergistic interactions between flavonols and green tea were confirmed.

These results suggested that the antioxidant synergistic effect between *O. fragrans* flowers and green tea can be attained through the accumulation of the synergistic effects of all the antioxidant compounds.

### Relative contributions of the antioxidant combinations to the antioxidant synergy in *O. fragrans* green tea

**Figure 4:**
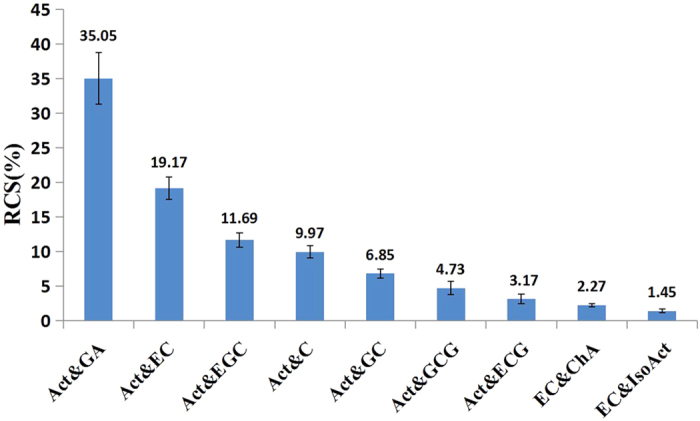
Relative contributions of the antioxidant combinations to the antioxidant synergy in *O. fragrans* green tea. RCS is the relative contribution of the antioxidant pair to the total antioxidant synergy between the *O. fragrans* flowers and green tea. The figure showed the major antioxidant pairs whose relative contribution higher than 1% in the order of RCS. Numerical results of RCS of all combinations were shown in Table S6.

Figure 4 showed the major antioxidant pairs (relative contribution higher than 1%) that contributed to the antioxidant synergy between *O. fragrans* flowers and green tea. The combination of acteoside and gallic acid had the most contribution (35.05%) to the antioxidant synergy, followed by other pairs, namely acteoside and EC (19.17%), acteoside and EGC (11.69%) and acteoside and catechin (9.97%). The combination of acteoside and gallic acid had the most contribution after simulated digestion because gallic acid was the major product of the decomposition of tea polyphenols, such as ECG and EGCG, which were extremely unstable in neutral or alkaline conditions^26^. Furthermore, acteoside had the highest content among the compounds in *O. fragrans* flowers, and gallic acid had the highest content among the compounds in green tea. Their combination had an RUV value of 0.54%, which mostly contributed to the antioxidant synergy between *O. fragrans* and green tea.

Acteoside was present in all the antioxidant pairs. It had high relative contributions because it had the highest content in the *O. fragrans* flowers. Acteoside isolated from several plants, such as *Premna serratifolia*^27^ and *Euphrasia rostkoviana*^28^, had also been shown to account for the major antioxidant effects in the *in vitro* assay models.

### Effect of simulated digestion on the antioxidant synergy between *O. fragrans* and green tea and their antioxidant compounds

A significant decrease in the antioxidant synergy between *O. fragrans* flowers and green tea after the simulated digestion was observed. Particularly, 32 RUV values of the 45 antioxidant combinations decreased after the simulated digestion (Table 2). Of these combinations, 15 decreased by more than 0.5%. Among the 15 combinations, 7 decreased by more than 1%. By contrast, the combinations where the RUV value increased slightly after simulated digestion did not increase by more than 0.5%.

The decrease of synergy resulted from the loss of the antioxidant compounds; moreover, their antioxidant capacities decreased (Table 1). Most phenylethanoid glycosides, such as acteoside, were typically hydrolysed or enzymolysed during simulated digestion^29^. Acteoside, which was present in all antioxidant pairs with high relative contributions, was reported to be unstable at pH 7, wherein it transformed into isoacteoside and other oxidative products; thus, its antioxidant synergy with other antioxidant compounds decreased. Similarly, some ester-type catechins, such as EGCG and ECG, undergo complex reactions during digestion to form EC and a EGCG derivative with high molecular weight, which has no antioxidant capacity^20^. Therefore, the relative contribution of the combination of acteoside and EGCG and that of acteoside and ECG to the total antioxidant synergy significantly decreased after the simulated digestion.

In conclusion, the general antioxidant synergistic effects among the antioxidant compounds accumulated and thus resulted in the antioxidant synergy between *O. fragrans* flowers and green tea. Among all the combinations among the antioxidant compounds, the combination of acteoside and gallic acid was a major antioxidant pair that had the most contribution to the antioxidant synergy of *O. fragrans* and green tea. However, the simulated digestion decreased the antioxidant synergy between *O. fragrans* flowers and green tea because of the decrease in the contents, antioxidant capacities and antioxidant synergies among the antioxidant compounds. Our research provided new insight into the *O. fragrans* green tea and its antioxidant synergistic effect and thus may assist in the future design of functional food or ingredients based on synergistic interactions.

## Materials and Methods

### Plant material and chemicals

*O. fragrans* flowers were collected at Lin’an (Zhejiang, China) in October 2014. The fresh flowers were dried by microwave vacuum drying combined with vacuum freeze drying. The dried flowers were then pulverised and sieved (20–40 mesh) for further use. Longjing Tea and Pu’er Tea were purchased from Hangzhou (Zhejiang, China); Keemun Black Tea was purchased from Qimen (Anhui, China); and Tieguanyin Tea was purchased from Anxi (Fujian, China).

Acteoside, isoacteoside, chlorogenic acid, salidroside, caffeic acid, catechin, gallic acid, caffeine, (−)-Epicatechin gallate (ECG), (−)-Gallocatechin gallate (GCG), (−)-Epigallocatechin gallate (EGCG), L-Epicatechin (EC), (−)-Epigallocatechin (EGC), (−)-gallocatechin (GC) and 2, 2-diphenyl-1-picrylhydrazylradical (DPPH) were obtained from Aladdin Industrial Co. (Shanghai, China). Caffeine was obtained from Sbjbio Co. (Nanjing, China). All other chemicals and reagents were of analytical grade.

### Sample preparation

The *O. fragrans* flowers and teas were extracted using a modified method described by Xiong *et al*.^30^. Approximately, 1 g of the dried sample was added to 45 mL boiling water. The mixture was then stirred and extracted at 55 °C for 0.5 h. The extract was then filtered and diluted to 50.0 mL in a brown volumetric flask and stored in a refrigerator at 4 °C for the evaluation of the antioxidant activities within 30 days.

The simulated digestion was performed using a modified method described by Versantvoort *et al*.^31^. Saliva with α-amylase was added to a buffer system with sodium and potassium at pH of 6.8 ± 0.2. Gastric juice containing pepsin was added to another buffer system with sodium, potassium and calcium at a final pH of 2.00 ± 0.02. Lastly, duodenal juice containing parenzyme and lipase was added to a buffer system at pH 8.1 ± 0.2.

The prepared extracts (1 g each) were incubated with 3 mL of saliva for 5 min and then were mixed with 6 mL of gastric juice for 120 min. The resulting mixture was then mixed with 6 mL of duodenal juice for 120 min. All the incubations were performed at 37 °C on a rotating wheel. After the digestion, ethanol was added to ensure the inactivation of the enzymes. The mixtures were then filtered by a vacuum pump. The filtrates were concentrated at 45 °C and then diluted to 10 mL with methanol. A controlled trial was operated without the prepared samples to improve the accuracy. The digesta of the extracts and antioxidant compounds were stored at 4 °C for the evaluation of the antioxidant activities within 30 days.

### Identification of the antioxidant compounds by LC-MS

The antioxidant compounds present in the *O. fragrans* tea were identified using a modified method described by Gruz *et al*.^32^. Analysis was performed on LC (ACQUITY, Waters, Milford, MA, USA) equipped with a mass spectrometre (Xevo TQ, Waters). The Zorbax XDB C18 column (2.1 mm × 150 mm, 3.5 μm) was maintained at 35 °C. Mobile phase A consisted of purified water with acetonitrile, whereas mobile phase B consisted of 0.1% formic acid (flow rate = 0.2 mL/min). The gradient elution profiles were as follows: 8% A for 0−3 min, from 8% to 12% A for 3–7 min, from 12% to 15% A for 7–11 min, from 15% to 20% A for 11–13 min, from 20% to 90% A for 17–19 min, from 90% to 8% A for 19–20 min and 8% A for 20–23 min. The injection volume was 1 μL. An ESI source was operated at negativation mode. The full-scan mass spectra ranged from 100 m/z to 1000 m/z. The parameters of the mass spectrometer were set at a capillary voltage of 3 (+) and 3.5 kV (−), capillary temperature of 325 °C, cone voltage of 135 V and sheath gas flow rate of 660 L/h^33^.

### Quantification of antioxidant compounds by UPLC-PDA

The antioxidant compounds present in the *O. fragrans* green tea were quantified using a modified method described by Trautvetter *et al*.^34^. The analysis was performed on UPLC (ACQUITY, Waters, Milford, MA, USA) equipped with a PDA detector (ACQUITY). The BEH-C18 symmetry column (150 mm × 2.1 mm, 1.7 μm) was maintained at 40 °C. Mobile phase A consisted of purified water with acetonitrile, whereas mobile phase B consisted of 85% phosphoric acid (flow rate = 0.2 mL/min). The gradient elution profiles were as follows: 8% A for 0−3 min, from 8% to 12% A for 3–7 min, from 12% to 15% A for 7–11 min, from 15% to 20% A for 11–13 min, from 20% to 90% A for 17–19 min, from 90% to 8% A for 19–20 min and 8% A for 20–23 min. The injection volume was 1 μL, and UPLC-PDA was set from 210.3 nm to 400 nm. The antioxidant compounds were quantified using the following standards: acteoside, salidroside, isoacteoside, caffeic acid, chlorogenic acid, catechin, gallic acid, EC, ECG, EGC, EGCG, GC, GCG and caffeine. The results were expressed as milligrams per gram of the dry weight.

### Determination of the antioxidant capacity

The antioxidant capacity was determined with DPPH antioxidant assay modified from the procedure developed by Sharma and Bhat^35^. The DPPH solutions were prepared in methanol (0.50 g/L) to provide an absorbance of ~1.2 units at 516 nm. The procedure was performed by adding 20 μL of the sample and 230 μL of the reagent into the wells (350 μL) of a microplate reader with 96 units (Thermo Scientific Nunc 96-Well Polypropylene MicroWell Plate with flat bottom). The reaction tubes, in triplicates, were wrapped in aluminum foil and kept at 30 °C for 60 min in dark. All the measurements were performed under dim light. Spectrophotometric measurements were conducted at 516 nm using Spectronic Genesys 5 spectrophotometer. Radical scavenging activity was expressed as the inhibition percentage and was calculated using the following formula:


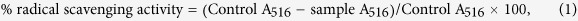


where A_516_ is the absorbance at 516 nm.

In each antioxidant, 15 increasing concentrations were used. In each concentration, 20 μL of sample was tested using the DPPH antioxidant assay^36^. The variation of the radical scavenging activity (R) as function of the dose of the antioxidant can be described satisfactorily using the Weibull cumulative distribution function^37^. Thus, the effect of increasing concentrations of an antioxidant (A) can be described in general terms, as follows:


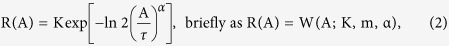


where *K* is the specific antioxidant asymptotic value of the response, m is the concentration producing the half-maximal response and α is a shape parameter related to the slope.

The parameter m of Eq. (2) directly provides the classical EC_50_ (μg of antioxidant), which effectively summarises all the effects (time and dose) of the response, providing the key information needed to achieve a very specific response (50%). In this instance, the lower the m value is, the higher the potency of antioxidant is.

### Determination of the synergistic and antagonistic effects

The antioxidant interaction was determined using a modified procedure developed by Prieto *et al*.^15^. The microplate assays were performed according to a complete design for 8 × 8 arrays of the two antioxidant mixtures at equally increasing concentrations (64 independent dose combinations), which were freshly prepared. Thus, 10 μL of each antioxidant solution was added to each well and was tested with the DPPH antioxidant assay. All the other conditions were the same as those previously described.

The dose response surface was fitted to the null interaction (NI) model


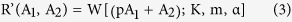


and the interaction (I) model


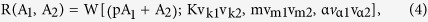


where p, v_k1_, v_k2_, v_m1_, v_m2_, v_α1_ and v_α2_ are additional fitting parameters.

An index that summarises the possible complex effects above was described to compute the percentage RUV between the surface volume (SV) produced by the null interaction and the surface volume with interactions, as follows:


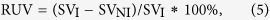


where SV is calculated by the integral of the dose response surface within the concentration determined.

### Relative contribution to synergism

The relative contribution of the antioxidants to the antioxidant synergy between *O. fragrans* flowers and green tea was calculated using the methods modified by Palafox-Carlos *et al*.^38^ and Lee *et al*.^39^. Firstly, the relative contribution of the antioxidant to the total antioxidant capacity of *O. fragrans* green tea (RCA) was calculated, as follows:


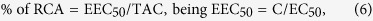


where EEC_50_ is the equivalent EC_50_ which is the content (C) of the antioxidant compound in *O. fragrans* tea divided by its EC_50_, which is the parameter m in Eq. (2); TAC is the total antioxidant capacity of the *O. fragrans* tea and is the sum of the EEC_50_ values of all the antioxidants present in *O. fragrans* tea.

Thus, for a certain pair of antioxidants, its relative RUV is as follows:


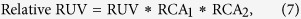


where RCA_1_ and RCA_2_ are the relative contributions of the antioxidant compounds to the total antioxidant capacity of *O. fragrans* tea.

In the end, the relative contribution of the antioxidant pair to the antioxidant synergy (RCS) between the *O. fragrans* flowers and green tea was calculated as follows:


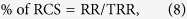


where RR is the relative RUV of the antioxidant pair; TRR is the total relative RUV of all the antioxidant pairs.

### Statistical analysis

All the experiments were performed in triplicate, and the results were expressed as mean ± SD. The simulated and experimental results were adjusted to the proposed models by nonlinear least squares methods (quasi-Newton) using Matlab 2013. Student’s t and Fisher’s F tests were used for model consistency. The statistically significant differences were expressed as *(P < 0.05) and **(P < 0.01).

## Additional Information

**How to cite this article:** Mao, S. *et al*. Antioxidant synergistic effects of *Osmanthus fragrans* flowers with green tea and their major contributed antioxidant compounds. *Sci. Rep.* **7**, 46501; doi: 10.1038/srep46501 (2017).

**Publisher's note:** Springer Nature remains neutral with regard to jurisdictional claims in published maps and institutional affiliations.

## Supplementary Material

Supplementary Material

## Figures and Tables

**Table 1:** Content and antioxidant capacity of antioxidant compounds in *O. fragrans* flowers and green tea before and after the simulated digestion.

Sample	Compound	Content(mg/g DW)		EC_50_(μg)	
		After	Before	After	Before
*O. fragrans* flowers	Act	10.77 ± 0.01	62.46 ± 0.82**	1.42 ± 0.08	0.89 ± 0.05**
	IsoAct	0.39 ± 0.00	2.86 ± 0.01**	1.07 ± 0.18	1.46 ± 0.16*
	ChA	0.27 ± 0.02	1.19 ± 0.06*	1.93 ± 0.15	0.81 ± 0.04**
	CA	0.08 ± 0.00	0.18 ± 0.00**	2.85 ± 0.21	0.55 ± 0.03**
	SAL	1.51 ± 0.03	4.60 ± 0.09*	/	/
Longjing Tea	C	0.54 ± 0.01	1.16 ± 0.01**	1.49 ± 0.08	1.42 ± 0.49
	CAF	10. 86 ± 0.45	23.01 ± 0.02*	/	/
	EC	1.82 ± 0.04	7.02 ± 0.08*	1.21 ± 0.05	0.88 ± 0.11**
	ECG	0.19 ± 0.03	1.78 ± 0.07**	1.96 ± 0.09	0.45 ± 0.05**
	EGC	1.12 ± 0.05	3.86 ± 0.09**	0.92 ± 0.04	0.51 ± 0.03*
	EGCG	nd	10.65 ± 0.92*	1.44 ± 0.10	0.98 ± 0.04*
	GA	2.06 ± 0.06	3.76 ± 0.09**	0.88 ± 0.07	1.54 ± 0.21**
	GC	1.14 ± 0.06	0.39 ± 0.05**	2.50 ± 0.12	1.23 ± 0.10**
	GCG	0.74 ± 0.13	3.30 ± 0.11**	0.99 ± 0.05	0.62 ± 0.06*

Act: actoside Verbascoside; IsoAct: isoacteoside; ChA: chlorogenic acid; CA: caffeic acid; SAL: salidroside; C: catechin; CAF: caffeine; EGCG: (−)-Epigallocatechin gallate; EC: L-Epicatechin; ECG: (−)-Epicatechin gallate; EGC: (−)-Epigallocatechin; GA: gallic acid; GC: (−)-gallocatechin; GCG: (−)-Gallocatechin gallate. Values are expressed as means ± SD (n = 3). nd: Not detected.

EC50: concentration of antioxidant for 50% of maximum effect. The concentration was showed in μg for the final reaction volume (250 μL). *Value and **Value was significantly different from that after simulated digestion at P < 0.05 and P < 0.01, respectively.

**Table 2:** Antioxidant interactive effects of 45 combinations of standard antioxidant compounds and extracts before and after the simulated digestion.

RUV(%)	Concentration range	Act (0–4.00)		IsoAct (0–4.00)		ChA (0–4.50)		CA (0–7.00)		*O. fragrans* (0–350.00)	
		After	Before	After	Before	After	Before	After	Before	After	Before
C	0–4.00	0.26	0.24	0.08	0.85	0.12	0.10	0.48*	0.99	0.11	0.30
EC	0–3.50	0.12	0.25	0.19	0.51	0.77*	0.36	0.42*	1.03	0.16	0.63
ECG	0–6.50	0.30	0.22	0.39*	0.72	0.65*	0.71	0.85*	1.88	0.01	0.24
EGC	0–3.00	0.09	0.22	0.06	0.12	0.50*	0.62	0.37*	0.22	−0.01	0.05
EGCG	0–5.00	0.05	0.07	0.12	0.06	0.51*	0.29	−0.47*	0.34	0.11	0.20
GA	0–2.00	0.54*	0.06	0.02	0.04	0.09	0.90	−0.36*	0.79	−0.12	0.27
GC	0–7.00	0.14	0.12	0.34*	2.21	0.75*	0.71	−0.21*	0.64	0.09	1.36
GCG	0–2.50	0.06	0.13	0.09	0.48	0.55*	0.60	0.31	0.68	0.43*	0.28
Tea	0–300.00	0.17	0.11	0.36*	0.28	0.37*	1.73	−0.59*	0.17	0.15	0.23

The concentration ranges used for each case were showed in μg for the final reaction volume (250 μL). The degree of antioxidant interactive effects (RUV) was computed as described in Eq. (5). *Value was significantly different from the control at P < 0.05. Each sample was combined with itself, and the results were used simply as a control.
