# Supplementary material for: Antioxidant synergistic effects of Osmanthus fragrans flowers with green tea and their major contributed antioxidant compounds
Source: Sci Rep. 2017 Apr 19;7:46501. doi: 10.1038/srep46501 (PMC5395974; doi:10.1038/srep46501)
Supplement: Supplementary Material [file srep46501-s1.doc]

**Antioxidant synergistic effects of *Osmanthus fragrans* flowers with green tea and their major contributed antioxidant compounds**

Shuqin Mao^1#^, Kaidi Wang^1#^, Yukun Lei^1^, Shuting Yao^1^, Baiyi Lu^1^*,Weisu Huang^1，2^*

1 [[1]](#footnote-2)Zhejiang University, College of Biosystems Engineering and Food Science, Fuli Institute of Food Science, Zhejiang Key Laboratory for Agro-Food Processing, Zhejiang R & D Center for Food Technology and Equipment, Key Laboratory for Agro-Food Risk Assessment of Minstry of Agriculture, Hangzhou 310058, China;

2 Zhejiang Economic & Trade Polytechnic, Department of Applied Technology, Hangzhou 310018, China

#These authors contributed equally to this work and should be considered co-first authors.

**Corresponding Author**:

*Dr. Baiyi Lu, E-mail address: bylu@zju.edu.cn .

*Dr. Weisu Huang, E-mail address: sophie212@163.com .

**Supplementary materials**

Table S.1 The differences among four kinds of traditional Chinese tea

| Features | | Longjing Tea | Pu’er Tea | Black Tea | Tieguanyin Tea |
| --- | --- | --- | --- | --- | --- |
| Variety | | unfermented tea | post-fermented tea | fully fermented tea | semi-fermented tea |
| Color | Tea leaf | green | black | red | totally green, with red fringes |
|  | Tea soup | green | brown-yellow or brown-red | red | orange |
| Production process | | fresh leaf, de-enzyme, rolling,  drying | fresh leaf,  de-enzyme, rolling,  pile fermentation, drying | fresh leaf, withering, rolling,  fermentation, drying | fresh leaf, withering, rotating,  de-enzyme, rolling,  drying |

*Table S.2 Parametric estimations and statistic information of the dose–responses of all antioxidants fitted to the Eq. (2) after simulated digestion*

| Antioxidants | Dose–effect parameters | | | Statistics |
| --- | --- | --- | --- | --- |
|  | K | m | α | R^2^_adj_ |
| Act | 0.90±0.02 | 1.42 ±0.08 | 1.63±0.17 | 0.9949 |
| IsoAct | 0.90±0.04 | 1.07±0.18 | 1.14±0.29 | 0.9717 |
| ChA | 0.91±0.03 | 1.93±0.15 | 1.94±0.36 | 0.9874 |
| CA | 0.92±0.05 | 2.85±0.21 | 1.59±0.22 | 0.9914 |
| C | 0.90±0.02 | 1.49±0.08 | 1.66±0.19 | 0.9950 |
| EC | 0.89±0.02 | 1.21±0.05 | 1.53±0.12 | 0.9973 |
| ECG | 0.90±0.02 | 1.96±0.09 | 1.37±0.11 | 0.9970 |
| EGC | 0.88±0.02 | 0.92±0.04 | 1.45±0.11 | 0.9973 |
| EGCG | 0.90±0.03 | 1.44±0.10 | 1.33±0.16 | 0.9934 |
| GA | 0.91±0.54 | 0.88±0.07 | 1.96±0.37 | 0.9869 |
| GC | 0.92±0.03 | 2.50±0.12 | 1.51±0.13 | 0.9967 |
| GCG | 0.90±0.02 | 0.99±0.05 | 1.66±0.18 | 0.9956 |
| *O. fragrans* | 0.99±0.13 | 122.80±24.02 | 1.19±0.24 | 0.9869 |
| Tea | 0.91±0.03 | 58.77±4.02 | 1.03±0.10 | 0.9958 |

*Table S.3 Parametric estimations and statistic information of the dose–responses of all antioxidants fitted to the Eq. (2) before simulated digestion*

| Antioxidants | Dose–effect parameters | | | Statistics |
| --- | --- | --- | --- | --- |
|  | K | m | α | R^2^_adj_ |
| Act | 0.84*±*0.03 | 0.89±0.05 | 1.61±0.19 | 0.9938 |
| IsoAct | 0.88*±*0.08 | 1.46±0.16 | 1.52±0.24 | 0.9912 |
| ChA | 0.88*±*0.02 | 0.81±0.04 | 1.51±0.13 | 0.9966 |
| CA | 0.90±0.01 | 0.55±0.03 | 1.63±0.16 | 0.9963 |
| C | 0.96±0.23 | 1.42±0.49 | 1.10±0.20 | 0.9898 |
| EC | 0.89±0.06 | 0.88±0.11 | 1.63±0.40 | 0.9716 |
| ECG | 0.88*±*0.03 | 0.45±0.05 | 1.25±0.20 | 0.9833 |
| EGC | 0.88±0.02 | 0.51±0.03 | 1.44±0.17 | 0.9943 |
| EGCG | 0.94±0.03 | 0.98±0.04 | 1.36±0.10 | 0.9976 |
| GA | 1.01±0.11 | 1.54±0.21 | 1.51±0.26 | 0.9897 |
| GC | 0.91±0.04 | 1.23±0.10 | 1.40±0.28 | 0.9910 |
| GCG | 0.89±0.03 | 0.62±0.06 | 1.69±0.35 | 0.9835 |
| O. fragrans | 0.94±0.10 | 12.03±1.79 | 1.30±0.25 | 0.9854 |
| Tea | 0.95±0.06 | 13.31±1.06 | 1.36±0.13 | 0.9964 |

K: the maximum radical reduced, asymptotic value of the response. m: the parameter corresponds to the dose required for 50% radical reduction (substrate half-dose). α: the shape parameter related to the maximum slope of the response. R^2^_adj_: correlation coefﬁcient adjusted between observed and predicted values.

Table S.4 The correlation coefficient of the joint action between different antioxidants by fitting the experimental results to the Eq. (4)

| R^2^_adj_ | Act | | IsoAct | | ChA | | CA | | *O. fragrans* | |
| --- | --- | --- | --- | --- | --- | --- | --- | --- | --- | --- |
|  | After | Before | After | Before | After | Before | After | Before | After | Before |
| C | 0.9591 | 0.9832 | 0.9864 | 0.9938 | 0.9890 | 0.9918 | 0.9924 | 0.9909 | 0.9687 | 0.9924 |
| EC | 0.9706 | 0.9876 | 0.9921 | 0.9904 | 0.9890 | 0.9854 | 0.9942 | 0.9948 | 0.9730 | 0.9929 |
| ECG | 0.9727 | 0.9853 | 0.9883 | 0.9952 | 0.9845 | 0.9901 | 0.9923 | 0.9944 | 0.9887 | 0.9882 |
| EGC | 0.9808 | 0.9852 | 0.9775 | 0.9924 | 0.9884 | 0.9864 | 0.9884 | 0.9917 | 0.9910 | 0.9948 |
| EGCG | 0.9885 | 0.9871 | 0.9916 | 0.9918 | 0.9842 | 0.9929 | 0.9864 | 0.9850 | 0.9931 | 0.9891 |
| GA | 0.9678 | 0.9817 | 0.9879 | 0.9894 | 0.9872 | 0.9886 | 0.9631 | 0.9870 | 0.9803 | 0.9906 |
| GC | 0.9890 | 0.9809 | 0.9910 | 0.9879 | 0.9893 | 0.9842 | 0.9890 | 0.9914 | 0.9835 | 0.9907 |
| GCG | 0.9855 | 0.9858 | 0.9887 | 0.9912 | 0.9901 | 0.9785 | 0.9975 | 0.9939 | 0.9900 | 0.9888 |
| Tea | 0.9796 | 0.9868 | 0.9960 | 0.9807 | 0.9831 | 0.9852 | 0.9611 | 0.9831 | 0.9941 | 0.9864 |

R^2^_adj_: correlation coefﬁcient adjusted between observed and predicted values.

Table S.5 Relative contribution of antioxidant compounds to the total antioxidant capacity of *O. fragrans* green tea

| Antioxidants | Content（mg/g） | EC50 | EEC50 | RCA(%) |
| --- | --- | --- | --- | --- |
| Act | 10.77±0.01 | 1.42±0.08 | 7.56±0.40 | 51.01±3.16 |
| IsoAct | 0.39±0.00 | 1.07±0.18 | 0.36±0.06 | 2.43±0.41 |
| ChA | 0.27±0.03 | 1.93±0.15 | 0.14±0.01 | 0.94±0.07 |
| CA | 0.08±0.00 | 2.85±0.21 | 0.03±0.00 | 0.20±0.01 |
| C | 0.54±0.01 | 1.49±0.08 | 0.36±0.02 | 2.43±0.16 |
| EC | 1.82±0.04 | 1.21±0.05 | 1.50±0.07 | 10.12±0.57 |
| ECG | 0.19±0.03 | 1.96±0.09 | 0.10±0.02 | 0.67±0.14 |
| EGC | 1.12±0.05 | 0.92±0.04 | 1.22±0.07 | 8.23±0.54 |
| EGCG | 0.00±0.00 | 1.44±0.10 | 0.00±0.00 | 0.00±0.00 |
| GA | 2.06±0.06 | 0.88±0.07 | 2.35±0.19 | 15.86±1.38 |
| GC | 1.14±0.06 | 2.50±0.12 | 0.46±0.03 | 3.10±0.23 |
| GCG | 0.74±0.13 | 1.00±0.05 | 0.74±0.14 | 4.99±0.96 |

RCA: the relative contribution of the antioxidant to the total antioxidant capacity, calculated as Eq. (6).

EC_50_: concentration for 50% of maximal effect.

EEC_50_: the equivalent EC_50_ which is the content of the antioxidant in the extract divided by its EC_50_.

Table S.6 Relative contribution of antioxidant pairs to the total antioxidant synergy between *O. fragrans* flowers and green tea

| RCS (%) | Act | IsoAct | ChA | CA | Sum |
| --- | --- | --- | --- | --- | --- |
| C | 9.97±0.90 | 0.15±0.03 | 0.08±0.01 | 0.07±0.01 | 10.28±0.90 |
| EC | 19.17±1.61 | 1.45±0.26 | 2.27±0.21 | 0.26±0.02 | 23.15±1.64 |
| ECG | 3.17±0.69 | 0.20±0.05 | 0.13±0.03 | 0.04±0.01 | 3.53±0.69 |
| EGC | 11.69±1.06 | 0.37±0.07 | 1.20±0.12 | 0.19±0.02 | 13.45±1.06 |
| EGCG | 0.00±0.00 | 0.00±0.00 | 0.00±0.00 | 0.00±0.00 | 0.00±0.00 |
| GA | 35.05±3.74 | 0.24±0.05 | 0.42±0.05 | -0.35±0.04 | 35.36±3.75 |
| GC | 6.85±0.66 | 0.79±0.15 | 0.68±0.07 | -0.04±0.00 | 8.28±0.68 |
| GCG | 4.73±0.96 | 0.34±0.09 | 0.80±0.16 | 0.10±0.02 | 5.96±0.97 |
| Sum | 90.65±4.51 | 3.53±0.32 | 5.57±0.31 | 0.26±0.05 | 100.00 |

RCS: the relative contribution of the antioxidant pairs to the total antioxidant synergy between the *O. fragrans* flowers and green tea, calculated as Eq. (8).

1. ^Z^ [↑](#footnote-ref-2)
